# *Ixodes scapularis* STING promotes Powassan virus infection in ticks

**DOI:** 10.21203/rs.3.rs-8620180/v1

**Published:** 2026-01-28

**Authors:** Xiu-Qi Tian, Dakota N. Paine, Alejandro Marín López, Dong Feng, Shanshan Du, Sukanya Narasimhan, Saravanan Thangamani, Erol Fikrig

**Affiliations:** 1 Section of Infectious Diseases, Department of Internal Medicine, School of Medicine, Yale University, New Haven, CT 06510, USA; 2 SUNY Center for Vector-Borne Diseases, Department of Microbiology and Immunology, Institute for Global Health and Translational Science, Upstate Medical University, Syracuse, NY 13210, USA; 3 Center of Research in Animal Health (CISA-INIA, CSIC), Algete-El Casar road, km. 8.1, Madrid 28130, Spain.; 4 These authors contributed equally

## Abstract

The stimulator of interferon genes (STING) is a central adaptor in antiviral signaling, but its role in vectors that transmit human and animal pathogens remains poorly understood. Here, we show that the tick STING homolog, tSTING, facilitates Powassan virus (POWV) infection in *Ixodes scapularis* -- in contrast to the canonical antiviral activity of mammalian STING. Transcriptomic and functional analyses revealed that tSTING regulates N-linked glycosylation and endocytosis, pathways essential for viral entry. Silencing of *RPN2*, a key glycosylation enzyme, significantly reduced viral loads, establishing a mechanistic link between tSTING and glycosylation-mediated viral uptake. Moreover, parallel studies in human THP-1 cells suggest human STING (hSTING) displays an opposite phenotype, restricting POWV replication potentially through OAS1-associated antiviral mechanisms independent of glycosylation. Together, these findings reveal an evolutionary reversal of STING function between arthropods and mammals, redefining the evolutionary logic of antiviral immunity across vector–host boundaries.

## Introduction

Arthropod vectors transmit a remarkable diversity of microbes that threaten human and animal health worldwide. In North America, the black-legged tick *Ixodes scapularis* spreads bacterial, protozoan, and viral pathogens, including the neuroinvasive Powassan virus (POWV)^1^. The molecular mechanisms, however, that allow ticks to tolerate persistent microbial colonization while remaining competent vectors are largely undefined.

Tick immunity relies largely on evolutionarily conserved innate pathways such as Toll, IMD, and JAK/STAT signaling^2–4^, which have been linked to responses against a range of microbial infections. However, these pathways alone do not fully explain how ticks can stably harbor and transmit multiple pathogens across their lifespan without apparent pathology. This discrepancy suggests that ticks may utilize additional immune strategies distinct from the classical pathogen-elimination mechanisms described in vertebrates.

The cyclic GMP–AMP synthase–stimulator of interferon genes (cGAS–STING) pathway is an evolutionarily conserved cytosolic nucleic acid sensing system found across bacteria^5–7^, invertebrates^8,9^, and vertebrates. In mammals, cGAS recognizes double-stranded DNA (dsDNA) and generates cGAMP to activate STING, triggering type I interferon (IFN-I) and interferon-stimulated gene (ISG) expression, as well as the transcription of genes encoding proinflammatory cytokines^10,11^. Despite its deep evolutionary origin, the cGAS–STING axis is not functionally uniform across species. cGAS-like enzymes differ in ligand selectivity and can sense either dsDNA or dsRNA^12^, producing distinct cyclic dinucleotides that activate diverse downstream effector programs^8,9 13,14^. This evolutionary flexibility raises the possibility that cGAS–STING has been repeatedly rewired to meet the immune demands of different organisms. Whether ticks encode a functional cGAS–STING pathway—and, if so, what biological role it serves—remains completely unknown.

Here, we identify cGAS–STING homologs in *I. scapularis* and uncover an unexpected role of tick STING (tSTING) in supporting POWV infection. *tSTING* silencing significantly lowered the acquisition of virus from an infected host, and transcriptomic analysis revealed that tSTING controls N-glycosylation and endocytic pathways required for viral entry. In mammalian cells, however, human STING (hSTING) restricted POWV replication without affecting glycosylation, instead correlating with increased expression of the antiviral effector OAS1. Thus, STING has undergone an evolutionary role reversal: in ticks, it acts as a proviral factor that supports viral acquisition, whereas in mammals it functions as an antiviral restriction molecule. This rewiring of an ancient immune module reveals how vector-borne viruses exploit conserved signaling architecture to persist in arthropods while being suppressed in vertebrate hosts.

## Results

### Identification of cGAS-STING homologs in *I. scapularis*

**Extended Data Fig.1.**
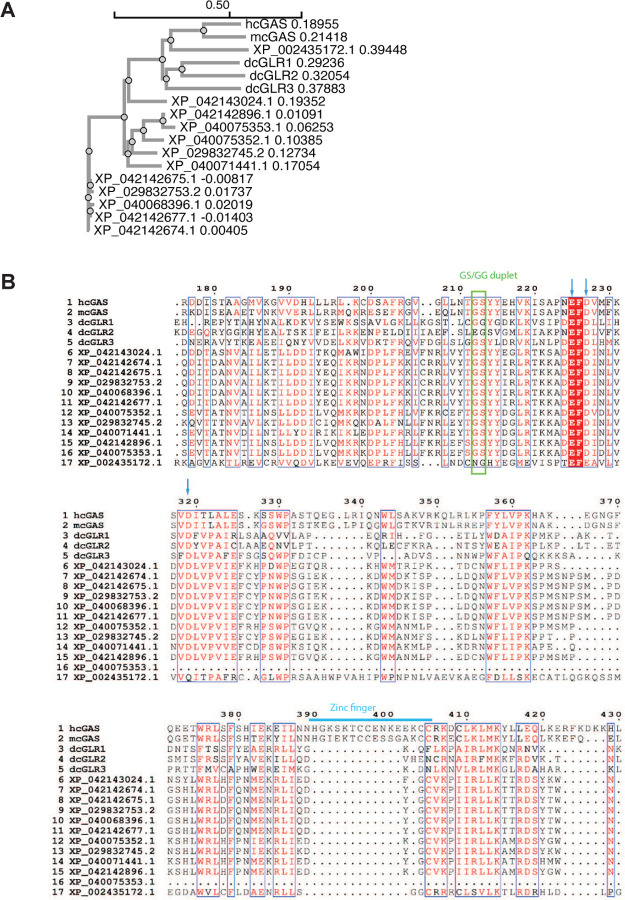
cGAS homologs in *Ixodes scapularis*. **(A)** Phylogenetic tree analysis of cGAS. The phylogenetic tree includes 12 tick cGAS homologous proteins (tcGAS) along with human cGAS (hcGAS), mouse cGAS (mcGAS), and *Drosophila* cGAS-like proteins (dcGLR1, dcGLR2, and dcGLR3). The tcGAS proteins were identified through NCBI-BLAST analysis, and the phylogenetic tree was constructed using Clustal Omega. **(B)** Partial sequence alignments of tcGAS proteins and other cGAS proteins are shown. The essential active site residues, including the GS/GG duplet, are highlighted within a green rectangle, while the metal ion-coordinating acidic residues are indicated by blue arrows. The Zn-ribbon of hcGAS and mcGAS are marked with a blue line.

To assess whether the cGAS–STING pathway is conserved in ticks, we searched for cGAS homologs in *I. scapularis*. BLASTP analysis using human, mouse, and *Drosophila* cGAS sequences identified 12 cGAS-like proteins (tcGAS) in the tick genome. Phylogenetic analysis showed that, except for XP_002435172.1 - which clusters with vertebrate cGAS - most tcGAS proteins form distinct invertebrate-specific branches (Extended Data Fig. 1A). Sequence alignment revealed that the majority of tcGAS proteins retain key catalytic features, including the GS/GG duplet and metal ion-coordinating acidic residue^9^ (Extended Data Fig. 1B), although none contain Zn-ribbon, consistent with other invertebrate cGAS homologs^12,15^.

**Extended Data Fig.2.**
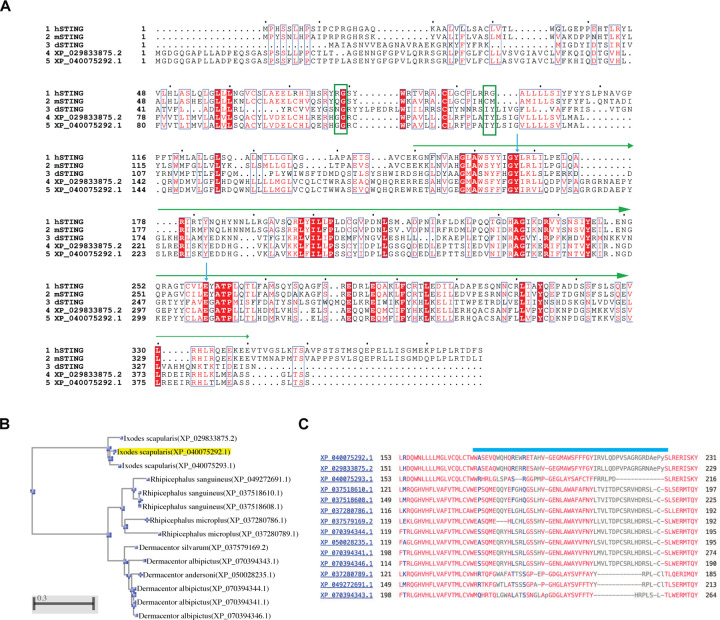
STING homologs among tick species. **(A)** Sequence alignments of tSTING and other STING proteins. The sequences of human STING (hSTING), mouse STING (mSTING), and *Drosophila* STING (dSTING) were obtained from the UniProt website. XP_029833875.2 and XP_040075292.1 are two tSTING variants in *I. scapularis*. The cyclic dinucleotide-binding domain (CBD) is highlighted with a green line, and the residues Tyr and Glu, which are proposed to be critical for cyclic dinucleotide binding, are marked with blue arrows. Black arrows indicate NS2B/3 cleavage sites in hSTING. **(B)** The phylogenetic tree of STING homologs across ticks was constructed using the neighbor-joining method based on NCBI-BLAST results. **(C)** Partial CBD sequence alignments of STING homologs across ticks. The regions with significant variation are highlighted with a blue line.

**Fig. 1.**
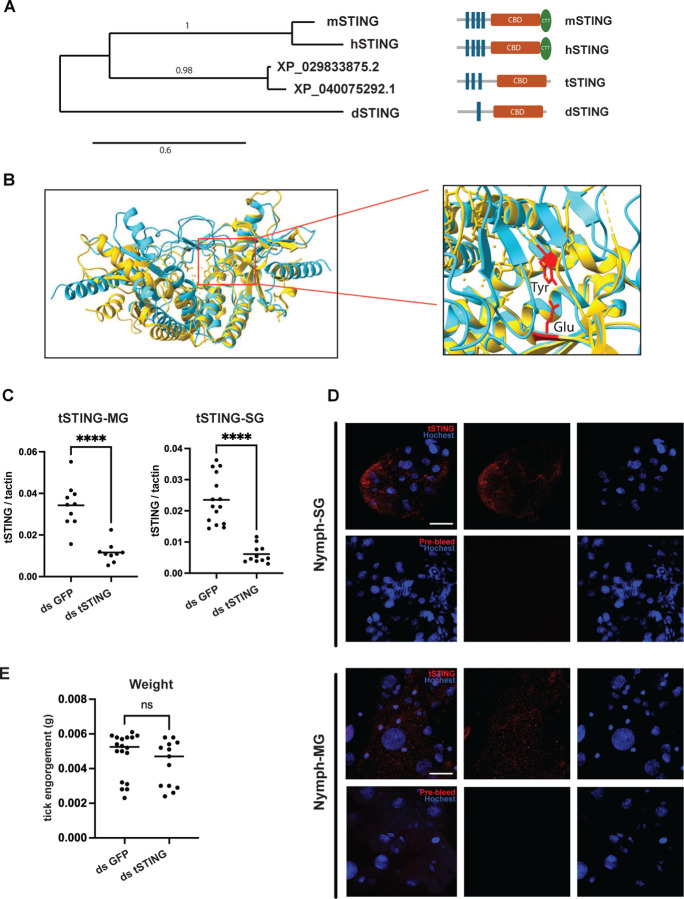
STING homologs in *Ixodes scapularis*. **(A)** Maximum-likelihood tree of STING proteins from mouse (mSTING, *Mus musculus*), human (hSTING, *Homo sapiens*), tick (tSTING, *Ixodes scapularis*; XP_029833875.2 / XP_040075293.1), and fly (dSTING, *Drosophila melanogaster*) generated using Phylogeny.fr (left). Domain schematics (right) indicate transmembrane segments, the cyclic-dinucleotide–binding domain (CBD), and the C-terminal tail (CTT). **(B)** The AlphaFold-predicted structure of tSTING-CBD-dimer (blue) is compared with the crystal structure of the hSTIN-GCBD-dimer complexed with 2’3’ cGAMP (yellow). The structural model of the tSTING dimer was obtained from the AlphaFold server, while the structure of hSTING complexed with 2’3’-cGAMP was sourced from the Protein Data Bank (PDB ID: 4LOH). Residues Tyr and Glu, associated with cGAMP binding, are highlighted in red. **(C)** Knockdown efficiency of *tSTING* in MG and SG following dsRNA treatment, quantified by qRT–PCR, normalized to β-actin. **(D)** Immunofluorescence staining of nymphal SG and MG using a rabbit polyclonal antibody raised against recombinant tSTING-CBD (red). Nuclei were counterstained with Hoechst (blue). Pre-bleed serum was used as a negative control. Scale bar = 25μm. **(E)** Tick engorgement weights following ds*GFP* or ds*tSTING* treatment. Each dot represents an individual tick. Statistical significance was assessed using a nonparametric Mann–Whitney test (ns, p>0.05; **p<0.01; ***p<0.001; ****p<0.0001).

We next searched for STING homologs using human, mouse, and *Drosophila* STING protein sequences. Two STING-like proteins (XP_040075292.1 and XP_029833875.2) were identified in *I. scapularis*, both sharing low sequence identity with vertebrate STINGs (~26–29%) but displaying 92% similarity to each other. No STING homolog was detected when *Drosophila* STING was used as a query, underscoring the divergence of tick STING from insect orthologs. Both proteins encode three transmembrane helices and a conserved cyclic-dinucleotide binding domain (CBD), but lack the C-terminal tail (CTT), a defining feature of invertebrate STINGs (Fig. 1A–B, Extended Data Fig. 2A). The essential Tyr and Glu residues required for cyclic dinucleotide (CDN) binding are preserved in both variants (Extended Data Fig. 2A). In addition, an AlphaFold model of the tick STING CBD dimer closely aligns with the crystal structure of the human STING CBD bound to 2′3′-cGAMP, with strong structural conservation within the ligand-binding pocket (Fig. 1B). A third isoform (XP_040075293.1) lacking one of the Glu residues was excluded from further analysis.

STING homologs were also detected in multiple additional tick species, including *Dermacentor silvarum*, *Dermacentor andersoni*, *Rhipicephalus microplus*, and *Rhipicephalus sanguineus* (Extended Data Fig. 2B), indicating that STING is broadly conserved across ticks. However, alignment of CBD domains revealed substantial sequence variation among species (Extended Data Fig. 2C), suggesting potential differences in CDN recognition or immune signaling across tick lineages.

Given the high redundancy of cGAS homologs and the presence of only two closely related STING homologs in *I. scapularis*, we focused our functional studies on the tick STING protein (hereafter referred to as tSTING). Because XP_040075292.1 and XP_029833875.2 share >90% sequence identity, we designed RNAi and qRT-PCR assays targeting the conserved region common to both homologs.

### Expression profile of tSTING and its impact on tick physiology

To determine whether tSTING is present in tissues involved in pathogen acquisition, we examined its expression at both the transcript and protein levels. tSTING was detected in the salivary glands (SG) and midguts (MG) of both nymphs and adults—two tissues critical for blood feeding and microbe transmission (Fig. 1C). We further confirmed the presence of tSTING protein by immunofluorescence staining using a rabbit polyclonal antibody generated against the recombinant tSTING-CBD protein (Fig. 1D).

To assess the physiological contribution of tSTING, we performed RNAi-mediated silencing by delivering dsRNA into the anal pore of nymphal ticks. qRT-PCR confirmed a robust reduction of tSTING mRNA following treatment (Fig. 1C). Despite efficient knockdown, *tSTING* silencing had no detectable effect on engorgement weight (Fig. 1E). We then examined whether it plays a role in microbial acquisition and transmission.

### tSTING enhances Powassan virus acquisition by ticks

**Fig. 2.**
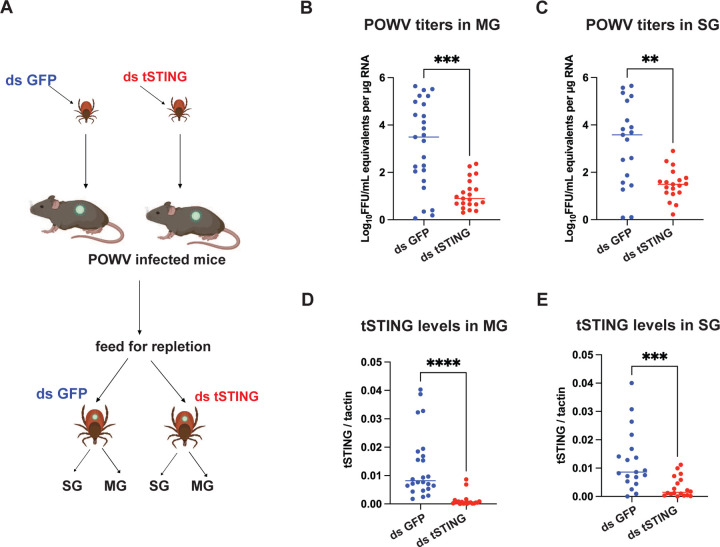
tSTING knockdown reduces POWV acquisition in ticks. **(A)** Experimental design. Nymphal ticks were injected with dsRNA targeting GFP (control) or tSTING, then allowed to feed to repletion on POWV-infected mice. SG and MG were dissected five days post-feeding for RNA extraction. **(B–C)** POWV RNA levels in SG (B) and MG (C) quantified by qRT-PCR and expressed as log_10_ FFU/mL equivalents per μg RNA. **(D–E)** Verification of *tSTING* knockdown in SG (D) and MG (E) by qRT-PCR, normalized to actin. Each dot represents an individual tick. Statistical significance was assessed using a nonparametric Mann–Whitney test (ns, p>0.05; **p<0.01; ***p<0.001; ****p<0.0001)

STING is classically characterized as an antiviral adaptor, particularly in the context of DNA virus sensing in mammals, but emerging evidence indicates that STING can also participate in RNA virus restriction^8,9,16^. The most clinically relevant virus transmitted by *I. scapularis* is Powassan virus (POWV), a positive-sense RNA flavivirus that causes severe encephalitis in humans. Whether tSTING contributes to antiviral defense or plays a distinct role during POWV infection remains unknown. To determine whether tSTING influences POWV acquisition, we performed RNAi knockdown in naïve nymphal ticks and allowed them to feed on POWV-infected mice (Fig. 2A). qRT-PCR analysis confirmed efficient *tSTING* silencing (Fig. 2D–E), and viral quantification revealed a marked reduction in POWV RNA levels in both MG and SG of *tSTING*-deficient ticks compared with controls after 5 days post drop off (Fig. 2B–C). Thus, instead of restricting infection, tSTING facilitates viral persistence in the tick vector.

### tSTING does not influence *Borrelia burgdorferi* acquisition by ticks or transmission from mice

We then determined whether the proviral function of tSTING extends beyond POWV to another medically important tick-borne pathogen, *Borrelia burgdorferi*, the causative agent of Lyme disease. Unlike viruses, *B. burgdorferi* is an extracellular spirochete, yet prior studies have shown that mammalian and *Drosophila* STING can restrict both intracellular^17,18^ and extracellular bacteria^19^ through cyclic-dinucleotide sensing or downstream antibacterial programs^20,21^.

**Extended Data Fig.3.**
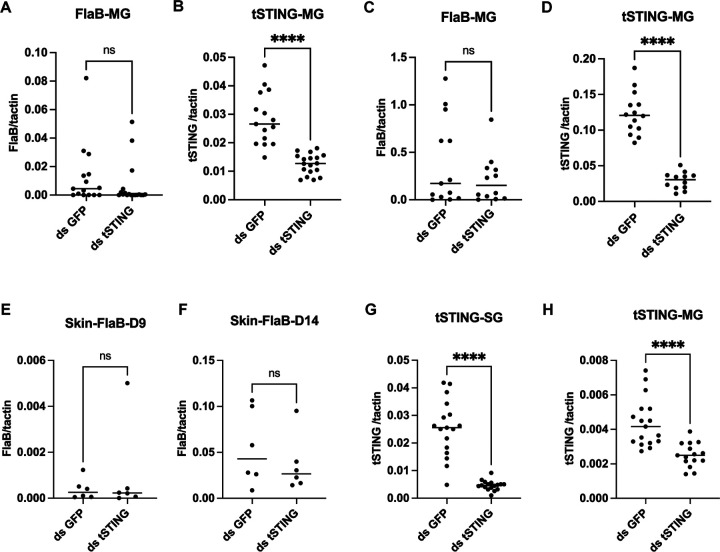
tSTING does not influence *B. burgdorferi* acquisition and transmission **(A-B)** *B. burgdorferi* acquisition at day 1 post-tick detachment. **(C-D)** *B. burgdorferi* acquisition at day 7 post-tick detachment. Each data point represents one tick. **(E-H)** *B. burgdorferi* transmission from ticks to mice. Mice skin samples were collected at day 9 and 14 post-tick attachment. Statistical significance was assessed using a nonparametric Mann–Whitney test (ns, p>0.05; **p<0.01; ***p<0.001; ****p<0.0001)

To determine whether tSTING modulates *B. burgdorferi* acquisition, we silenced *tSTING* in naïve nymphs and allowed them to feed on infected mice. Quantification of the spirochete burden in the midgut at day 1 and day 7 post-feeding revealed no difference between *tSTING*-silenced and control ticks (Extended Data Fig. 3A–D). We further tested whether tSTING affects bacterial transmission to mammalian hosts by silencing *tSTING* in *B. burgdorferi*-infected nymphs prior to feeding on naïve mice. Spirochete loads in the skin at days 9 and 14 post-attachment were indistinguishable between groups (Extended Data Fig. 3E–H), indicating that tSTING has no detectable impact on transmission efficiency.

Together, these results demonstrate that, unlike its striking proviral effect on POWV, tSTING does not influence the acquisition or transmission of *B. burgdorferi*. This selective requirement for viral - but not bacterial - pathogen colonization suggests that tSTING has evolved a virus-specific role in the tick vector rather than a broad antimicrobial function.

### tSTING rewires glycosylation and endocytic pathways during POWV infection

**Extended Data Fig.4.**
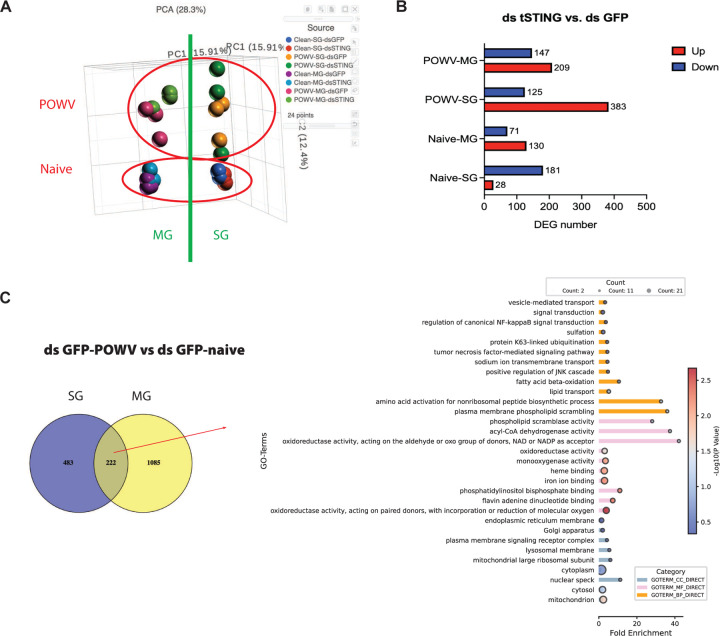
Overall description of RNA-Seq data in naïve ticks and POWV-infected ticks. **(A)** Principal component analysis (PCA) was performed on all normalized data to identify patterns and clusters. Distinct patterns and clusters are highlighted with red circles and green line. The analysis was conducted using Partek Flow. **(B)** Differentially expressed genes (DEGs) were identified when comparing ds *tSTING* with ds *GFP*. DEGs with a log2 fold change (log2FC) value of 1 were enriched for further analysis. The lists of these DEGs were generated using Partek Flow. **(C)** Venn diagram showing differentially expressed genes (DEGs) identified by comparing POWV-infected ticks with naïve ticks under control (dsGFP) conditions. The right bar plot indicates the Gene Ontology (GO) terms and KEGG pathways enriched among the 222 common genes identified between SG and MG.

Because ticks lack an interferon system and tSTING exhibits a proviral phenotype, the canonical STING–IFN antiviral model cannot explain its function in ticks. To define the mechanism by which tSTING promotes POWV infection, we profiled tSTING-dependent transcriptional programs in SG and MG from both naïve and POWV-infected ticks. Principal component analysis (PCA) revealed clear separation by tissue type and infection state (Extended Data Fig. 4A), confirming the robustness of the transcriptomic dataset. Differentially expressed genes (DEGs) were defined as those exhibiting ⩾1.5-fold change (up- or down-regulated) with FDR < 0.05. To delineate transcriptional responses driven by POWV infection independently of tSTING, we compared dsGFP-POWV versus dsGFP-naïve samples. This analysis identified 705 DEGs in SG and 1,307 DEGs in MG, of which 222 genes were shared between the two tissues (Extended Data Fig. 4C). GO enrichment analysis of these shared genes revealed that POWV infection prominently altered genes involved in vesicle-mediated transport, lipid and phospholipid metabolism, and host signaling pathways such as NF-κB and JNK. Enriched molecular functions were largely related to oxidoreductase and lipid-binding activities, while cellular component terms were dominated by organelle-associated membranes including the ER, Golgi, mitochondria, and plasma membrane signaling complexes. Together, these results indicate that POWV reshapes host metabolic and membrane-trafficking pathways to create a cellular environment conducive to viral replication.

**Extended Data Fig.5.**
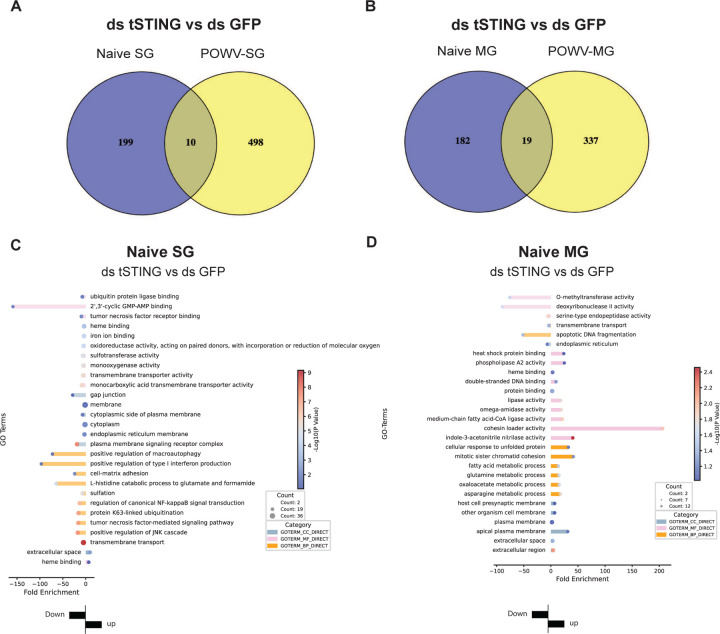
tSTING-regulated genes in naïve SG and MG. **(A)** The Venn diagram displays the overlap of tSTING-regulated genes between naïve SG and POWV-SG. A total of 10 genes are common to both conditions. **(B)** The Venn diagram displays the overlap of tSTING-regulated genes between naïve MG and POWV-MG. A total of 19 genes are common to both conditions. **(C)** The bar plot displays the GO terms and KEGG pathways of genes regulated by tSTING in naïve salivary glands (SG). In the plot, the left columns represent downregulated genes, while the right columns represent upregulated genes. **(D)** The bar plot displays the GO terms and KEGG pathways of genes regulated by tSTING in naïve midguts (MG). In the plot, the left columns represent downregulated genes, while the right columns represent upregulated genes.

We next examined genes regulated by tSTING under each condition. Upon *tSTING* knockdown, naïve SG showed 28 upregulated and 181 downregulated genes, whereas naïve MG showed 130 up and 71 down. Following POWV infection, SG displayed 383 up and 125 down, and MG showed 209 up and 147 down (Extended Data Fig. 4B). Notably, there was minimal overlap in enriched genes between naïve and POWV-infected ticks across these tissues (Extended Data Fig. 5A–B), highlighting distinct differences in *tSTING*-regulated genes under naïve versus POWV-infected conditions.

To identify the DEGs regulated by tSTING in naïve ticks and POWV-infected ticks, we conducted GO term enrichment and KEGG pathway analyses. In naïve-SG (Extended Data Fig. 5C), the downregulated gene set after *tSTING* knockdown was enriched for oxidoreductase and monooxygenase activity, ion and transmembrane transport, and ER- and plasma membrane–associated components. These categories are broadly linked to redox balance, small-molecule movement across membranes, and local signaling at epithelial interfaces, which may reflect a basal contribution of tSTING to maintaining metabolic and membrane-associated homeostasis under non-infected conditions. In naïve MG (Extended Data Fig. 5D), *tSTING* knockdown resulted in the downregulation of a small set of genes enriched in O-methyltransferase activity, DNase II activity, serine-type endopeptidase activity, transmembrane transport, apoptotic DNA fragmentation, and endoplasmic-reticulum–associated functions. By contrast, the majority of enriched terms were upregulated and included amino-acid and fatty-acid metabolic processes (asparagine, glutamine, oxaloacetate, and lipid metabolism), unfolded-protein and heat-shock responses, and a range of catalytic activities such as phospholipase, lipase, CoA-ligase activity. Upregulated categories also extended to membrane and extracellular components, including the apical plasma membrane, presynaptic membrane, and extracellular region. Overall, the downregulated terms were confined to a narrow set of nuclease- and ER-related functions, whereas the upregulated categories spanned broader metabolic, proteostasis, and membrane-associated programs.

**Fig. 3.**
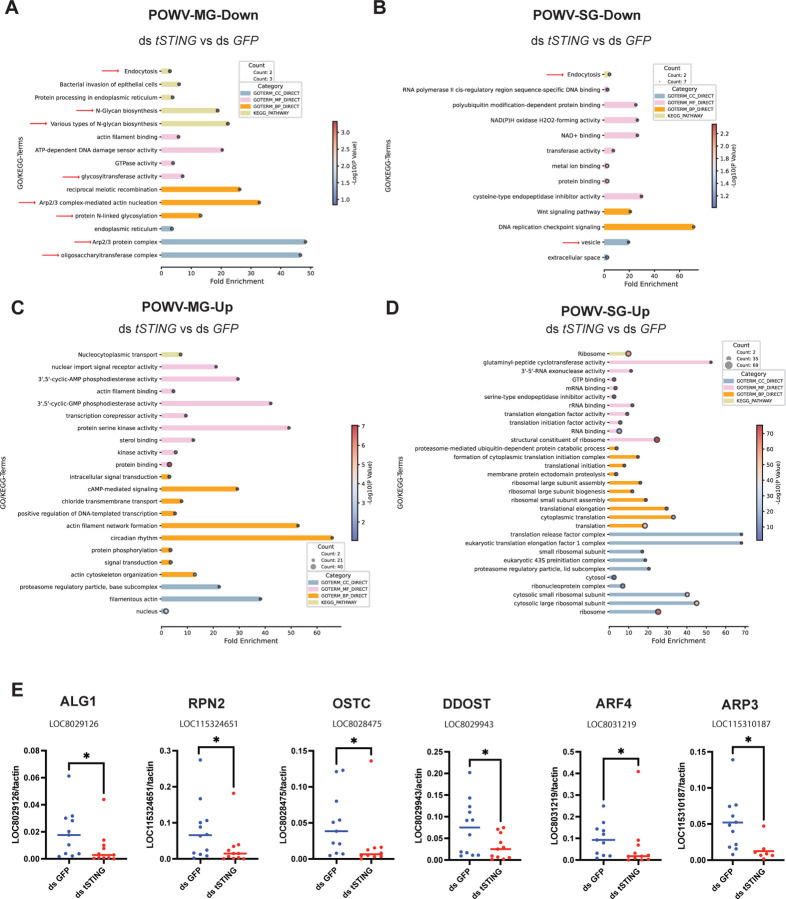
Transcriptomic analysis identifies tSTING-regulated pathways during POWV infection in ticks. **(A–D)** Functional enrichment analysis of differentially expressed genes (DEGs) in POWV-infected MG and SG following *tSTING* knockdown. (A) Downregulated DEGs in POWV-infected MG (POWV-MG–DOWN). (B) Downregulated DEGs POWV-infected in SG (POWV-SG–DOWN). (C) Upregulated DEGs in POWV-infected MG (POWV-MG–UP). (D) Upregulated DEGs in POWV-infected SG (POWV-SG–UP). Enriched categories include Gene Ontology (GO) terms (Biological Process [BP], Cellular Component [CC], Molecular Function [MF]) and KEGG pathways. The top significantly enriched terms (FDR < 0.05) are shown, ranked by fold enrichment. Dot size reflects the number of DEGs; bar color denotes annotation category. **(E)** qRT-PCR validation of selected *tSTING*-regulated genes in POWV-infected MG, including glycosylation-associated genes (ALG1, RPN2, OSTC, DDOST) and endocytosis/cytoskeleton-associated genes (ARF4, ACTR3). Transcript levels were normalized to actin. Each dot represents an individual tick. Statistical significance was assessed using a nonparametric Mann–Whitney test (*p < 0.05).

Because the overlap between tSTING-regulated genes in naïve and POWV-infected ticks was minimal (Extended Data Fig. 5A–B), we next focused on the transcriptional programs specifically controlled by tSTING during viral infection. In POWV-MG, *tSTING* knockdown led to significant downregulation of genes enriched in endocytosis, N-glycan biosynthesis, and ER protein processing. Enriched molecular functions included Arp2/3 complex assembly, actin filament binding, and GTPase activity (Fig. 3A), pointing to coordinated regulation of cytoskeletal dynamics and vesicle trafficking—processes essential for viral internalization. By contrast, the upregulated genes were enriched in nucleocytoplasmic transport, cyclic-nucleotide phosphodiesterase and kinase signaling, actin-linked cytoskeletal remodeling, proteasome regulatory subunits, and transcriptional control (Fig. 3C).

In POWV-SG, *tSTING* silencing led to marked downregulation of genes involved in endocytosis, vesicle-mediated transport, oxidoreductase activity, and metal-ion binding (Fig. 3B), again implicating membrane trafficking and redox regulation in *tSTING*-dependent viral support. In contrast, the 383 upregulated genes were strongly enriched in ribosome biogenesis and translation, including pathways related to ribosomal assembly and translation initiation/elongation (Fig. 3D), consistent with increased protein synthesis during viral replication.

Overall, endocytosis-associated genes were among the most consistently suppressed after *tSTING* knockdown, suggesting that tSTING supports POWV acquisition by sustaining the endocytic machinery required for virion entry. In addition to actin-dependent endocytosis^22–24^, flavivirus entry is known to require N-glycosylation of the viral envelope protein^25–28^, and may also be influenced by host cell–derived glycans that modulate attachment and internalization^29^. Thus, the simultaneous reduction of endocytosis- and glycosylation-related genes provides a plausible explanation for the decreased viral burden observed in *tSTING*-silenced ticks.

### Glycosylation genes downstream of tSTING are required for efficient POWV infection

**Fig. 4.**
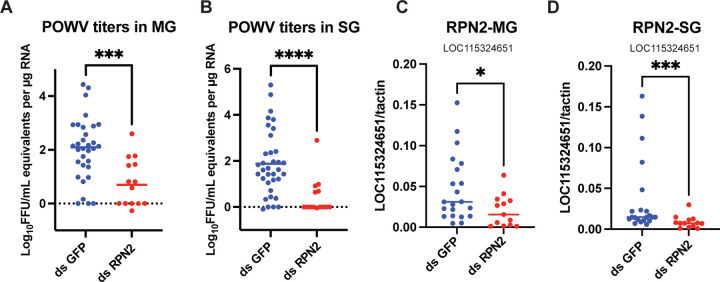
Interference with glycosylation reduces POWV infection in ticks. **(A)** Viral loads in MG of ticks injected with ds*GFP* or ds*RPN2*. **(B)** Viral loads in SG of the same ticks. Titers were quantified as log_10_ FFU/mL equivalents per μg RNA. Mann–Whitney test (***p < 0.001; ****p < 0.0001).

To validate the transcriptomic signatures identified above, we quantified the expression of representative tSTING-regulated genes by qRT-PCR. These included three genes involved in N-linked glycosylation—ALG1 (LOC8029126), RPN2 (LOC115324651), and OSTC (LOC8028475)—and two genes associated with vesicle transport and endocytosis—ARF4 (LOC8031219) and ARP3 (LOC115310187) (Extended Data Table 1). Consistent with the RNA-Seq results, all five genes were significantly downregulated upon *tSTING* silencing (Fig. 3E), confirming tSTING-dependent regulation of glycosylation and endocytic machinery. To directly test whether glycosylation is required for POWV infection in ticks, we silenced RPN2, a core subunit of the oligosaccharyltransferase (OST) complex essential for N-glycan assembly. RPN2 depletion led to a marked reduction of POWV burden in both MG and SG of ticks 5 days after drop-off (Fig. 4A–D), phenocopying the effect of *tSTING* knockdown. These results establish that RPN2 - and by extension - N-glycosylation is required for efficient viral acquisition in ticks. Together with the transcriptomic data, these findings position tSTING as a positive regulator of the glycosylation–endocytosis axis that enables POWV entry, rather than a classical antiviral signaling node.

### Human STING inhibits POWV infection in THP-1cells

**Fig. 5.**
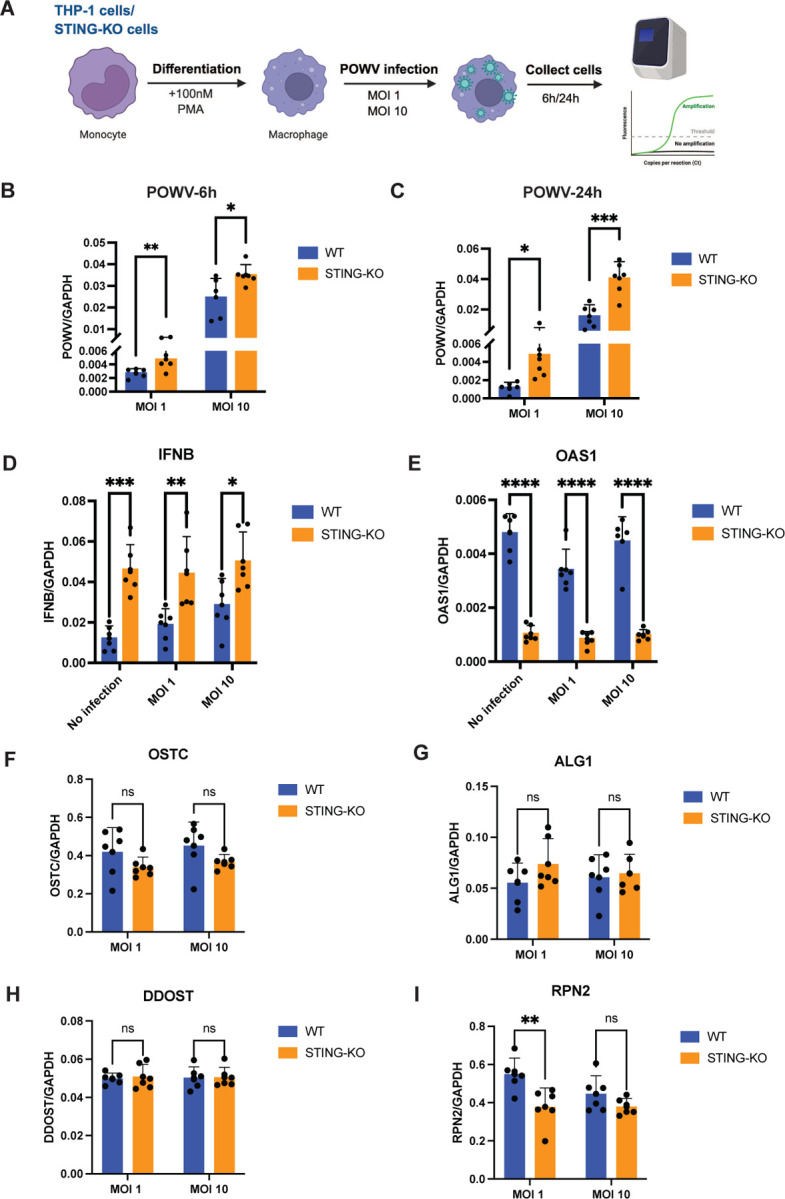
Human STING restricts POWV infection in THP-1 cells. **(A)** Schematic of the experimental workflow. THP-1 wild-type (WT) and STING-knockout (STING-KO) cells were differentiated into macrophage-like cells using 100 nM PMA for 24 h, followed by POWV infection at MOI 1 or MOI 10. Cells were collected at 6 h or 24 h post-infection for RNA extraction and qRT-PCR analysis. **(B–C)** Quantification of POWV RNA levels normalized to GAPDH at 6 h (B) and 24 h (C) post-infection. **(D–E)** qRT-PCR analysis of IFNB1 (D) and OAS1 (E) transcript levels in mock-infected or POWV-infected cells at 6 h post-infection. **(F–I)** qRT-PCR analysis of glycosylation-related genes OSTC (F), ALG1 (G), DDOST (H), and RPN2 (I) in POWV-infected cells at 6 h post-infection. For all panels, each dot represents a biological replicate; data were normalized to GAPDH. Statistical analysis was performed using an unpaired two-tailed t-test. ns, not significant; *P < 0.05; **P < 0.01; ***P < 0.001; ****P < 0.0001.

Since tSTING supports POWV infection in the tick vector, we next investigated whether its mammalian counterpart, human STING (hSTING), similarly promotes POWV infection or instead functions as a classical antiviral restriction factor. To address this, we examined POWV infection in wild-type (WT) and STING-knockout (STING-KO) THP-1 cells. Following PMA-induced differentiation, cells were infected with POWV at MOI 1 or 10, and viral RNA levels were quantified at 6 and 24 hours post-infection (Fig. 5A). In all conditions, viral RNA levels were markedly higher in STING-KO cells than in WT cells (Fig. 5B–C), indicating that hSTING exerts an antiviral effect against POWV infection. In addition, we determined whether this antiviral effect is mediated by Type I IFN (IFNB1) and interferon-stimulated genes (ISGs) at 6h in infected cells. Interestingly, the antiviral effect of hSTING does not appear to be mediated by IFN-β, as STING-KO cells exhibited higher basal levels of IFNB expression even in the absence of POWV infection (Fig. 5D). This observation suggests that alternative pathways may compensate for the loss of STING to induce IFNB expression. However, despite elevated IFNB levels, the interferon-stimulated gene OAS1 (which encodes a 2′-5′-oligoadenylate synthetase 1 critical for the innate antiviral response through the degradation of viral RNA^30–32^) was significantly downregulated in STING-KO cells (Fig. 5E). This effect also occurred even in the absence of POWV infection, indicating that STING is essential for maintaining the basal expression of OAS1. Our results therefore suggest that STING’s POWV antiviral effect may involve, at least in part, an OAS1-dependent mechanism.

Given that tSTING promotes POWV infection in ticks by upregulating glycosylation-dependent viral entry, we next asked whether hSTING also regulates the expression of these glycosylation-related genes. qRT-PCR analysis at 6h in infected cells revealed that expression of glycosylation-related genes—including OSTC, ALG1, and DDOST—was unchanged in STING-KO cells (Fig. 5F–H). Interestingly, RPN2 expression was instead downregulated in STING-KO cells at MOI 1 (Fig. 5I), further suggesting that hSTING does not influence POWV infection through glycosylation-mediated entry.

Together, these results reveal a mechanistic divergence in STING function across species: tSTING promotes POWV infection by enabling glycosylation-dependent viral entry in ticks, whereas hSTING restricts infection in human cells, potentially involving OAS1-linked antiviral activity.

## Discussion

**Fig. 6.**
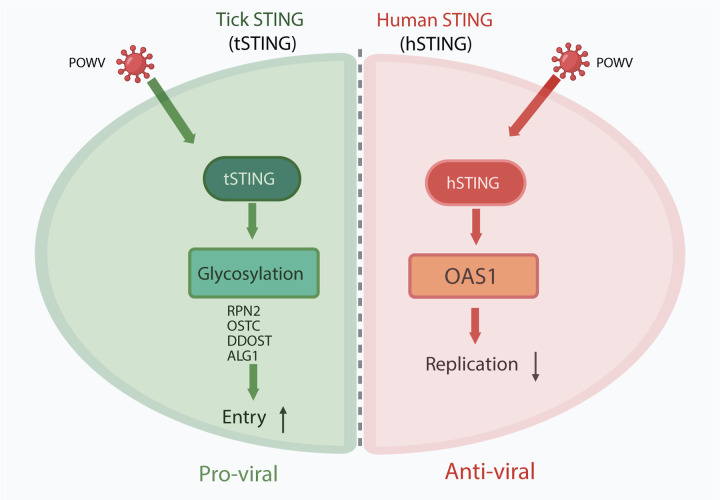
Model: Divergent roles of STING in tick and human antiviral responses This model illustrates the opposite functional outcomes of STING signaling in the tick vector versus the mammalian host during Powassan virus (POWV) infection. In *Ixodes scapularis*, tick STING (tSTING) acts as a pro-viral factor by transcriptionally upregulating N-linked glycosylation machinery (including RPN2, OSTC, DDOST, and ALG1), which enhances glycosylation-dependent viral entry and promotes productive infection. In contrast, human STING (hSTING) restricts POWV replication, potentially through OAS1-associated antiviral activity, independent of glycosylation pathways. Thus, while STING activation in mammals limits viral replication, the same pathway in ticks is rewired to facilitate viral persistence, revealing an evolutionary reversal in STING function across vector–host boundaries.

In this study we uncover a functional divergence in the cGAS–STING axis between arthropod vectors and mammalian hosts (Fig. 6): tick STING (tSTING) promotes POWV infection (Fig. 2), whereas human STING (hSTING) maintains its canonical antiviral role and limits infection in THP-1 cells (Fig. 5). Mechanistically, tSTING enhances viral entry by transcriptionally upregulating N-glycosylation and endocytosis-associated pathways, altering the expression of genes like ALG1, RPN2 and OSTC, and thereby facilitating virion internalization in tick cells (Fig. 3–4). In striking contrast, hSTING does not modulate glycosylation but instead supports antiviral defense—potentially via the OAS1–RNase L axis—despite elevated type I interferon levels upon STING loss (Fig. 5). These data establish an evolutionary reversal in STING function between arthropods and mammals, revealing how a conserved immune adaptor can be co-opted to enable persistent viral colonization in vector tissues while continuing to restrict infection in vertebrate hosts.

Intriguingly, STING is conserved in ticks yet completely absent in major mosquito vectors^33^, such as *Aedes* mosquitoes and *Anopheles* mosquitoes. This stark phylogenetic divergence suggests that different arthropod vectors have arrived at distinct solutions to balance antiviral defense with microbial tolerance. While mosquitoes rely on fast RNA interference (RNAi)^34^, Toll^35^, and JAK–STAT pathways^36^ for antiviral defense to clear acute infections, ticks retain STING — likely because it fulfills essential physiological or immune roles required for their long lifespan, prolonged blood-feeding, and sustained interactions with diverse microbes. Our findings further suggest that POWV opportunistically exploits this pre-existing STING-regulated program, reflecting how a conserved innate module can be co-opted to facilitate viral persistence in a long-lived vector.

While STING is typically antiviral in mammals, an exception exists. Human rhinoviruses (RVs) have been reported to hijack mammalian STING to replication organelles to enhance viral replication^37–39^. However, this mechanism differs from what we observed in ticks. tSTING promotes POWV infection by regulating glycosylation-related pathways that facilitate viral entry. This distinction underscores a previously unrecognized connection between innate immune signaling and host cell glycosylation machinery.

**Extended Data Fig.6.**
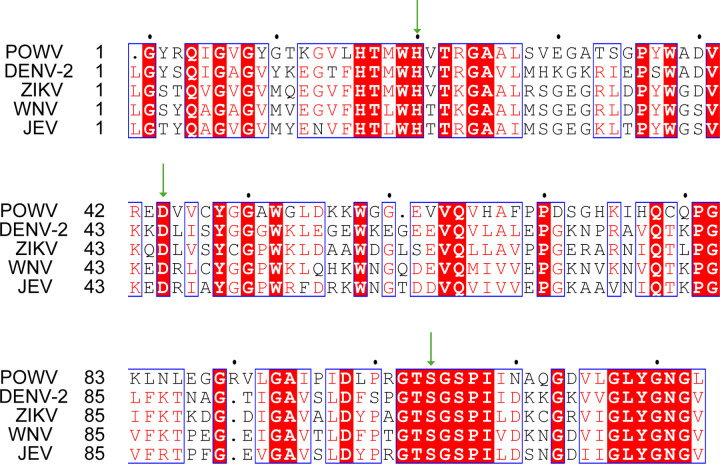
Sequence alignment highlighting the conserved catalytic triad in flavivirus NS3 proteases. Partial alignment of the NS3 protease regions from representative flaviviruses—Dengue virus 2 (DENV-2), Zika virus (ZIKV), West Nile virus (WNV), Japanese encephalitis virus (JEV), and Powassan virus (POWV)—performed using Clustal Omega. The canonical catalytic residues His^51^, Asp^75^, and Ser^135^ (numbering based on DENV-2 NS3) are indicated by green arrows, showing strict conservation across all examined flaviviruses. Sequence logo representation of the aligned region further illustrates the high conservation of the catalytic triad within the protease active site.

Although STING typically restricts flaviviruses in human cells, flaviviruses such as ZIKV and DENV can proteolytically degrade hSTING via their NS2B/3 proteases to subvert its antiviral role^40–42^. The presence of conserved NS2B/3 cleavage motifs in POWV (Extended Data Fig. 6) raises the possibility that it may employ a similar strategy in mammalian hosts. In contrast, tSTING lacks these conserved cleavage sites, including the RG motifs at residues 78/79 and 96/97 (Extended Data Fig. 2A), suggesting that POWV may selectively antagonize hSTING while sparing tSTING. This dichotomy is consistent with a bifurcated host–vector strategy, whereby POWV antagonizes STING-mediated immunity in vertebrates while co-opting tSTING-dependent glycosylation pathways in its arthropod vector.

In conclusion, our work uncovers a unique and evolutionarily intriguing reversal of STING function -- acting as a proviral factor in ticks while maintaining an antiviral role in mammals. This finding suggests that POWV has evolved to utilize tSTING-dependent pathways to establish persistent infection within the tick vector, supporting efficient maintenance and transmission between arthropods and vertebrates. An important unresolved question concerns how the cGAS–STING pathway is activated in ticks. Ticks encode multiple cGAS-like proteins, yet whether these enzymes generate noncanonical cyclic dinucleotides capable of engaging tSTING, or whether tSTING instead signals through a CDN-independent mechanism, remains unknown. Elucidating the endogenous ligands and activation circuitry of the tick cGAS–STING axis will be critical for understanding how ancient innate immune modules are evolutionarily repurposed to support durable virus tolerance in arthropod vectors.

## Materials and methods

### Ethics Statement

All the experiments involving C3H/HeN mice and *B. burgdorferi*-infected mice in this study were performed in accordance with the Guidelines for the Care and Use of Laboratory Animals of the National Institutes of Health, USA. The animal protocols were approved by the Institutional Animal Care and Use Committee at the Yale University School of Medicine. All experiments involving POWV-infected BALB/C mice and ticks were conducted in an arthropod containment level 3 (ACL-3) facility in accordance with an animal use protocol approved by the State University of New York (SUNY) Upstate Medical University Institutional Animal Care and Use Committee (IACUC).

### RNA extraction, cDNA synthesis and quantitative reverse transcription polymerase chain reaction (qRT-PCR) based assays

To evaluate gene expression of target genes in ticks, pathogen-free or infected fed nymphs were dissected under a dissecting microscope to obtain midguts (MG) and salivary glands (SG). The RNA from dissected MG and SG was purified using RLT lysis buffer according to the manufacturer’s protocol (QIAGEN), and cDNA was synthesized using the iScript cDNA Synthesis Kits (Bio-Rad, #1708891). qRT-PCR was performed using an iTaq Universal SYBR Green Supermix (Bio-Rad, # 1725121) with an initial denaturing step of 2 min at 95°C and 45 amplification cycles consisting of 20 s at 95°C followed by 15 s at 60°C, and 30 s at 72°C. The target genes and corresponding primer sequences are shown in Extended Data Table 2.

### Immunofluorescence staining

SG and MG from nymphal ticks were dissected 3 days after blood feeding and fixed in 4% paraformaldehyde (PFA) overnight at 4 °C. MG samples were additionally treated with ice-cold acetone for 5 min to enhance tissue permeability. Tissues were washed three times with PBS containing 0.1% Triton X-100 and blocked in 5% BSA with 0.1% Triton X-100 for 1 h at room temperature (RT). Samples were incubated overnight at 4 °C with rabbit anti-tSTING serum (1:1500; generated against recombinant tSTING-CBD), its corresponding pre-bleed serum as a negative control (1:1500). After three washes with PBS/0.1% Triton X-100, tissues were incubated with Alexa Fluor 488–conjugated anti-rabbit IgG (1:500; Invitrogen, A11008) for 2 h at RT. Nuclei were stained with Hoechst following the final PBST wash, and tissues were mounted on slides and coverslipped for imaging.

### Silencing of gene expression using dsRNA

Fed-nymph SG and MG cDNA was prepared as described above and used as template to amplify segment of target gene. Primers were designed by addition of a T7 promoter sequences (TAATACGACTCACTATAGGGAGA). Double-stranded RNA (dsRNA) was synthesized using the MEGAscript RNAi kit (Invitrogen, #AM1626M) using PCR-generated DNA template that contained the T7 promoter sequence at both ends. The dsRNA quality was examined by agarose gel electrophoresis. dsRNA of the Aequorea victoria green fluorescent protein (GFP) was used as a control. dsRNA (50 nL) was injected into the anal pore of nymphs using needles as described earlier^43^.

### Animals, ticks, spirochetes, and cell lines

Six-week-old female C3H/HeN mice were purchased from the Charles River Laboratories. Five-week-old female BALB/C mice were obtained from the Jackson Laboratory (Bar Harbor, ME). To obtain spirochetes-infected mice, the mice were injected subcutaneously with 100 μL of 1×10^5^ cells/mL *B. burgdorferi* (strain B31). Pathogen-free *I. scapularis* larvae were obtained from the Oklahoma State University (Stillwater, OK). The larval ticks were fed on pathogen-free C3H/HeN mice and allowed to molt to nymphs. To generate *B. burgdorferi*-infected nymphs, the larvae were placed on spirochetes-infected mice. All the ticks were maintained at 85% relative humidity with a 14h light and 10h dark period at 23°C. The spirochetes *B. burgdorferi* were grown in Barbour-Stoenner-Kelly H (BSK-H) complete medium (Sigma-Aldrich, #B8291) in a 33°C setting incubator. The live cell density was determined by dark field microscopy and hemocytometer (INCYTO, #DHC-N01). THP-1 wild-type cells were obtained from our laboratory cell bank. The STING-knockout THP-1 cell line was purchased from InvivoGen (Cat. code: thpd-kostg).

### Animal infections and tick infestation of virus

Five-week-old female BALB/C mice were obtained from The Jackson Laboratory (Bar Harbor, ME). They were given an acclimation period of one week prior to the experimental start time. The mice were then individually housed in ventilated cage systems alongside controlled temperature, humidity, and a 12-hour light-dark cycle. On Day 0, mice were anesthetized with isoflurane and oxygen and had their backs shaved to allow for capsule placement as previously described^44^. In brief, capsules were attached onto the backs of the shaved mice and secured with Kamar adhesive. On the same day, mice were inoculated with 10^4^ FFU (focus forming units) of POWV-LB strain via footpad injection. The following day, 10 nymphal ticks were placed into the capsules of each mouse and secured in place. For the following days of the study, nymphs were observed for attachment, and mice were observed for clinical signs and symptoms. Later in the study, nymphs were collected as they dropped off and upon euthanasia of the mouse, ticks were sorted into the groups based on the day they dropped off the mouse. After dropping off, fed ticks were housed in groups according to the day of drop off in an environmental chamber held at 22°C and 85–90% humidity. After 5 days post drop off, ticks were dissected and the salivary glands, and midgut for each tick was dissected and placed into RLT (Qiagen) buffer. RNA isolation using RNeasy 96 QIAcube HT kit was then performed on each sample and quantified using a DS-11+ spectrophotometer (DeNovix).

### Viral infection quantification

Infection titers for each sample were determined via qRT-PCR, using POWV primers (POWV-F: CGAGCCAAAGTGAGGATGT and POWV-R: TCTTTTGCCGAGCTCCACTT) as described previously^45^. The following cycling protocol was used with a CFX Opus 96 (BioRad): 10 min at 50°C; 1 min at 95°C; 10 s at 95°C followed by 30 s at 60°C for 45 cycles; and an 81-cycle (+0.5°C/cycle) 55°C −95°C melt curve.

### *B. burgdorferi* acquisition of ticks and transmission to the murine host

For *B. burgdorferi* acquisition experiment, C3H/HeN mice were subcutaneously injected with 100 μL of *B. burgdorferi* (B31) spirochetes (10^5^/mL)^46^. Two weeks post-injection ear skin biopsies were obtained from each animal and *B. burgdorferi* burden assessed by qPCR as described earlier. Comparably infected mice were challenged with approximately 20 pathogen free nymphal ticks and allowed to feed to repletion. MG was dissected at day 1 or day 7 after tick attachment and total RNA was isolated from each nymph. To assess transmission, 3 *B. burgdorferi*-infected nymphs were placed on each C3H/HeN mouse (5 mice used in each control or experimental group) and ticks were allowed to feed to repletion. *B. burgdorferi* burden in mice was also assessed as described in skin at day 9 and 14 post-tick attachment.

### RNA-seq analysis

Total RNA was extracted from salivary glands and midguts of naïve ticks and POWV-infected ticks. Libraries were generated using the Low-Input PolyA RNA Prep kit and sequenced on an Illumina HiSeq 2500 (paired-end) at the Yale Center for Genome Analysis (YCGA). Raw reads were trimmed and aligned to the *Ixodes scapularis* genome (PalLabHiFi assembly) using STAR (v2.7.8a). Gene-level counts were assigned to Ensembl release 100 annotations using the Partek E/M algorithm, followed by total count normalization with a pseudocount offset (0.0001)^46^. Principal component analysis (PCA) and heatmaps were generated in Partek Flow using default settings. Differentially expressed genes (DEGs) were defined as those showing ⩾1.5-fold change (up- or down-regulated) with FDR < 0.05. GO term and KEGG pathway enrichment analyses were performed using DAVID.

### THP-1 infection assay

THP-1 wild-type (WT) and STING-knockout (STING-KO) cells were cultured in RPMI-1640 supplemented with 10% FBS. Cells were differentiated into macrophage-like cells by treatment with 100 nM PMA for 24 h, followed by a 24 h rest period in PMA-free medium. Differentiated cells were infected with POWV at multiplicities of infection (MOI) of 1 or 10. Cells were harvested at 6 h or 24 h post-infection, and total RNA was extracted for qRT-PCR analysis of viral RNA and host gene expression.

### Sequence alignment of NS3 protease catalytic regions

Amino acid sequences corresponding to the catalytic region of the NS3 protease were retrieved from representative flaviviruses, including Dengue virus 2 (DENV-2, NP_739587.2), Zika virus (ZIKV, YP_009227202.1), West Nile virus (WNV, YP_001527884.1), Japanese encephalitis virus (JEV, NP_775670.1), and Powassan virus (POWV, NP_775520.1). Partial sequences encompassing the conserved catalytic motifs were aligned using Clustal Omega (EMBL-EBI) with default parameters. The alignment output was visualized in Jalview, and the catalytic triad residues—His^51^, Asp^75^, and Ser^135^ (numbering based on DENV-2 NS3)—were annotated and highlighted using green arrows to indicate their strict conservation across mosquito-borne and tick-borne flaviviruses.

## Extended Data

**Extended Data Table 1:** Functional annotation of genes analyzed by qRT-PCR

Gene ID	Name	Annotation	GO/KEGG
LOC8029126	ALG1	chitobiosyldiphosphodolichol beta-mannosyltransferase	Protein-N-linked glycosylation
LOC115324651	RPN2	Dolichyl diphosphooligosaccharide–protein glycosyltransferase subunit 2	Protein-N-linked glycosylation
LOC8028475	OSTC	Oligosaccharyltransferase complex subunit	Protein-N-linked glycosylation
LOC8031219	ARF4	ADP-ribosylation factor 4	Vesicle-mediated transport
LOC115310187	ARP3	actin-related protein 3	Endocytois/Arp2/3 complex-mediated actin nucleation
LOC8029943	DDOST	Dolichyl-diphosphooligosaccharide–protein glycosyltransferase 48 kDa subunit	Protein-N-linked glycosylation

**Extended Data Table 2:** Primer sequences in this study

Gene	Forward sequence	Reverse sequence
tSTING-qRT-PCR	GGAACCGTACTACTGCCT	TCTCTCCAGCAGCTCCTT
tSTING-dsRNA	TAATACGACTCACTATAGGGAGAAGCACATACAGGACACTG	TAATACGACTCACTATAGGGAGACGTACTTGCTGATGCGTT
Tactin-qRT-PCR	ACCTGACCGACTACCTGATG	CAGAGCTTCTCCTTGATGTCG
POWV-qRT-PCR	CGAGCCAAAGTGAGGATGT	TCTTTTGCCGAGCTCCACTT
FlaB-qRT-PCR	GAGTTCATGTTGGAGCAA	GGAGAATTAACTCCGCC
mactin-qRT-PCR	AGAGGGAAATCGTGCGTGA	CTGGGTACATGGTGGTACC
LOC115310187-qRT-PCR	ACGTCGTCCCCTCTACAAGA	GTGATACGGCCTCCACTCAG
LOC8029126-qRT-PCR	ACTGGACCTGCCCATGAAAG	TCTTCGCTCGACTGGAACAC
LOC115324651-qRT-PCR	CCCGTCTGCTCGGAAATACA	ACGCAACCACGTCCATTTTG
LOC8028475-qRT-PCR	GCATCGGATCTACGGTGGAC	CCGTGAAGAGGAAGCTCGAC
LOC8031219-qRT-PCR	CTACCAAATGCCATGCCTGC	GACAGTTCGTTGGAGAGCCA
LOC8029943-qRT-PCR	GAACTGGCGAACGAAGTTGG	CGGCAACTATGGTTGTGTGC
IFNB-qRT-PCR	CTTGGATTCCTACAAAGAAGCAGC	TCCTCCTTCTGGAACTGCTGCA
OAS1-qRT-PCR	AGGAAAGGTGCTTCCGAGGTAG	GGACTGAGGAAGACAACCAGGT
OSTC-qRT-PCR	CTGTTCATTGGATTCGTCTGTG	GCACTCTAACCCATCAGATAGC
ALG1-qRT-PCR	CTCAGAGGAACTGGCAGCTC	CACCCAGCTCTCATCCCATC
DDOST-qRT-PCR	ACTCCAGCACACGCAGTATG	CTCCTTCTCCTTCATGTGCAAG
RPN2-qRT-PCR	GGACTCAGCTCAACATGTTCC	TGTGCTGTTCTCTTGACTGC
GAPDH-qRT-PCR	GTCTCCTCTGACTTCAACAGCG	ACCACCCTGTTGCTGTAGCCAA
